# The Effects of Oral l-Arginine and l-Citrulline Supplementation on Blood Pressure

**DOI:** 10.3390/nu11071679

**Published:** 2019-07-22

**Authors:** David Khalaf, Marcus Krüger, Markus Wehland, Manfred Infanger, Daniela Grimm

**Affiliations:** 1 Department of Biomedicine, Aarhus University, 8000 Aarhus C, Denmark; 2 Clinic for Plastic, Aesthetic and Hand Surgery, Otto von Guericke University Magdeburg, 39120 Magdeburg, Germany; 3 Department of Microgravity and Translational Regenerative Medicine, Faculty of Medicine and Mechanical Engineering, Otto von Guericke University Magdeburg, 39120 Magdeburg, Germany

**Keywords:** amino acids, nutrition, nitric oxide, eNOS, blood pressure, hypertension

## Abstract

Nitric oxide (NO) is a well-known vasodilator produced by the vascular endothelium via the enzyme endothelial nitric oxide synthase (eNOS). The inadequate production of NO has been linked to elevated blood pressure (BP) in both human and animal studies, and might be due to substrate inaccessibility. This review aimed to investigate whether oral administration of the amino acids l-arginine (Arg) and l-citrulline (Cit), which are potential substrates for eNOS, could effectively reduce BP by increasing NO production. Both Arg and Cit are effective at increasing plasma Arg. Cit is approximately twice as potent, which is most likely due to a lower first-pass metabolism. The current data suggest that oral Arg supplementation can lower BP by 5.39/2.66 mmHg, which is an effect that is comparable with diet changes and exercise implementation. The antihypertensive properties of Cit are more questionable, but are likely in the range of 4.1/2.08 to 7.54/3.77 mmHg. The exact mechanism by which Cit and Arg exert their effect is not fully understood, as normal plasma Arg concentration greatly exceeds the Michaelis constant (K_m_) of eNOS. Thus, elevated plasma Arg concentrations would not be expected to increase endogenous NO production significantly, but have nonetheless been observed in other studies. This phenomenon is known as the “l-arginine paradox”.

## 1. Introduction

Hypertension is a state of elevated systolic and/or diastolic blood pressure. For years, blood pressure (BP) equal to or higher than 140/90 mmHg was classified as hypertension, but the 2017 updated guidelines from the American College of Cardiology and American Heart Association classified hypertension as BP equal to or higher than 130/80 mmHg [1]. An optimal blood pressure level is a reading under 120/80 mmHg. Hypertension is a known risk factor for many diseases, including stroke, myocardial infarction, and renal disease [2]. In 2015, the worldwide prevalence of hypertension was estimated to be 1.13 billion [3], and deaths attributable to this preventable risk factor have previously been estimated to 9.4 million per year worldwide [4]. Despite affecting more than a billion individuals worldwide, 95% of the cases do not present with known secondary causes of hypertension such as renal failure or pheochromocytoma, and therefore are termed essential hypertension [2].

BP is the product of cardiac output and total peripheral resistance, and an increase in one or both variables contributes to elevated BP. The regulation of BP is complex and is affected through various mechanisms, such as sympathetic nervous system activity, the renin–angiotensin–aldosterone system, and the nitric oxide (NO) pathway [5]. An in-depth description of the various mechanisms involved is beyond the scope of this communication, which instead will focus on the NO pathway in relation to l-arginine (Arg) and l-citrulline (Cit) supplementation. Arg is a semi-essential amino acid found in various foods, and is synthesized by the body itself. It serves as an intermediate in the urea cycle, and as a substrate for the synthesis of NO. Cit is a non-essential amino acid, and functions as an intermediate in the urea cycle as well. Through two subsequent enzymatic reactions, Cit is converted to Arg, and thus may serve as a precursor for the endogenous synthesis of Arg [6], which in theory could augment the synthesis of NO. NO is involved in the regulation of BP by inducing vasodilation via the stimulation of soluble guanylyl cyclase (sGC) and the subsequent rise in intracellular cyclic guanosine monophosphate (cGMP), which ultimately results in vasodilation [7].

The primary objective of this communication is to review the individual and/or combined effects of Cit and Arg on blood pressure, and whether these effects are of clinical importance in the treatment of hypertension. The initial literature search was performed on PubMed using the following search terms “Citrulline supplementation + hypertension” and “Arginine supplementation + hypertension” yielding 48 and 239 search results, respectively, as of 26 June 2019. Articles concerning pulmonary hypertension and preeclampsia were excluded, as these were deemed to be irrelevant. Additional relevant articles were identified in the reference list of the remaining articles and by using the “similar articles” and “cited by” function in PubMed. Later searches using the PubMed MeSH function with the input “(((“Arginine”[Mesh]) OR “Citrulline”[Mesh]) AND “Nitric Oxide”[Mesh]) AND “Hypertension”[Mesh])” restricted to reviews and clinical trials yielded 122 search results as of 26 June 2019, and additional relevant articles were identified in the same manner as previously described.

## 2. Nitric Oxide in Blood Pressure Regulation

### 2.1. Nitric Oxide-Mediated Vasodilation

Nitric oxide (NO) is an important gaseous signaling molecule in mammals (including humans) that is able to diffuse freely across membranes. NO has a half-life of only a few seconds, but it contributes to the regulation of cardiac contractility and dilates blood vessels [8,9,10], leading to increased blood supply and decreased BP [8]. Changes in the vascular endothelium, similar to a deficiency in the l-arginine–NO pathway, are involved in hypertension [11]. It also contributes to vessel homeostasis by inhibiting vascular smooth muscle contraction and growth. Humans suffering from hypertension, diabetes, or atherosclerosis often show impaired NO signaling pathways [12]. Inhaled NO has been shown to be a potent vasodilator [13]. Beyond cardiovascular regulation, NO is involved in numerous further physiological and pathophysiological processes [14]. NO is biosynthesized endogenously from Arg, O_2_, and nicotinamide adenine dinucleotide phosphate (NADPH) by three isoforms of nitric oxide synthase, which are termed endothelial (eNOS), neuronal (nNOS), and inducible (iNOS). The eNOS isoform is considered the most important isoform in relation to the cardiovascular system [14].

**Figure 1:**
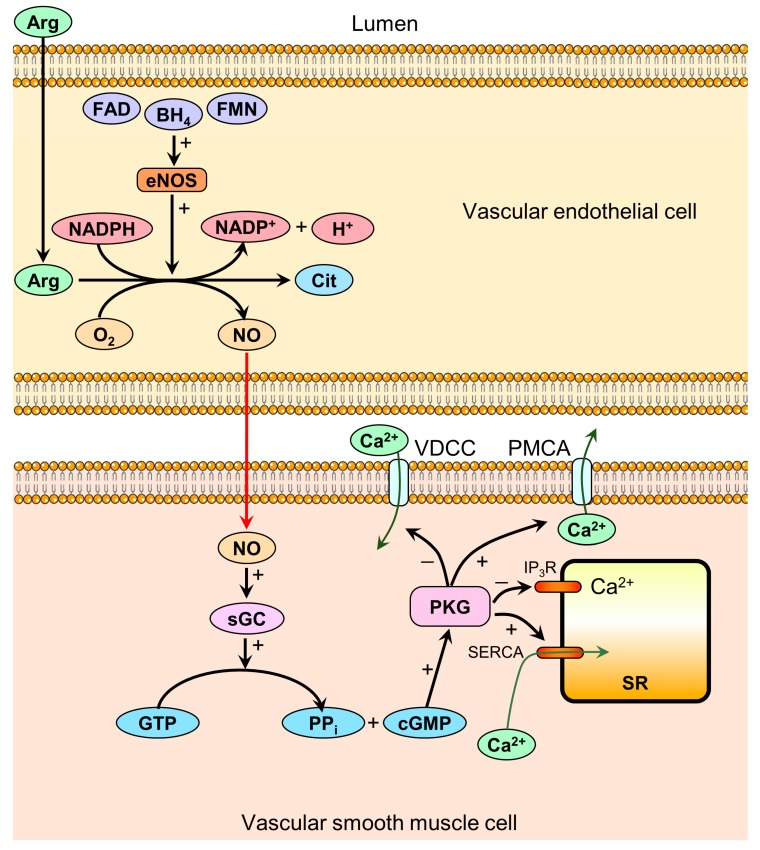
Mechanisms of nitric oxide-mediated vasodilation. Plasma Arg provides the substrate for the synthesis of nitric oxide (NO) via the enzyme endothelial nitric oxide synthase (eNOS) located in the vascular endothelium. The enzymatic reaction requires the co-substrates O_2_ and nicotinamide adenine dinucleotide phosphate (NADPH) and the cofactors BH_4_, flavin adenine dinucleotide (FAD), and flavin mononucleotide (FMN). NO diffuses from the endothelial cell to the smooth muscle cell and activates soluble guanylyl cyclase (sGC), resulting in increased cyclic guanosine monophosphate (cGMP) production. cGMP subsequently activates protein kinase G (PKG), resulting in decreased [Ca^2+^]_i_ via at least four mechanisms: 1. Inhibition of voltage-dependent calcium channels (VDCC), reducing calcium influx. 2. Activation of plasma membrane calcium ATPases (PMCA), increasing ATP-dependent calcium efflux. 3. Inhibition of inositol triphosphate receptors (IP_3_R), reducing calcium release from the sarcoplasmic reticulum (SR) to the cytoplasm. 4. Activation of sarcoplasmic calcium ATPases (SERCA), increasing the ATP-dependent sequestration of calcium from the cytoplasm to the SR. Decreased [Ca^2+^]_i_ mediates smooth muscle relaxation via the activation of myosin light chain kinase and the inhibition of myosin light chain phosphatase (not shown in figure), resulting in vasodilation.

As the name implies, eNOS is expressed in the vascular endothelium, and is responsible for the synthesis of NO, which subsequently diffuses to the underlying vascular smooth muscle cells. In these cells, NO activates sGC, which catalyzes the conversion of guanosine triphosphate (GTP) to cGMP and inorganic pyrophosphate (PP_i_) [15]. cGMP activates protein kinase G (PKG), resulting in decreased intracellular calcium concentration ([Ca^2+^]_i_) via at least four separate mechanisms (Figure 1) [16]. The reduced [Ca^2+^]_i_ prevents the activation of myosin light chain kinase by the calmodulin–calcium complex, and furthermore increases the activity of myosin light chain phosphatase, resulting in smooth muscle relaxation [7].

The significance of eNOS mediated vasodilation was demonstrated in mice with disruption in the eNOS gene, resulting in a mean BP 20 mmHg higher than the wild-type mice in the awake state [17]. Furthermore, human studies have shown an inverse relationship between average mean daytime BP and urinary nitrate excretion (used as a measure of endogenous NO production) and that nitrate excretion was lower in patients with untreated essential hypertension compared to healthy controls [18]. This suggests that impaired NO production might be implicated in the pathogenesis of essential hypertension, even though the authors could not determine the direction of causality. Rats treated with the NOS inhibitor, N^G^-nitro-l-arginine-methyl ester (L-NAME), developed hypertension [19]. Schlaich et al. [20] used the intra-arterial infusion of radiolabeled Arg and venous blood sampling to demonstrate reduced Arg uptake in hypertensive and normotensive males with a positive family history of hypertension compared with normotensive males with a negative family history, which could result in an inability to maintain sufficient intracellular Arg concentrations for NO synthesis. Furthermore, these two groups were the only groups who improved forearm blood flow during the co-administration of acetylcholine and Arg, compared with only acetylcholine, suggesting that Arg supplementation could improve acetylcholine-mediated vasodilation in these patient groups. These findings by Schlaich et al. [20] were not due to any significant difference in mRNA or protein expression of the main endothelial Arg transporter, CAT-1, which was assessed via real-time PCR in mononuclear cells, nor due to different asymmetric dimethylarginine (ADMA) levels (a competitive endogenous inhibitor of eNOS) between the three groups, with the latter finding possibly due to the small sample size (*n* = 42). The authors concluded that Arg availability might be a rate-limiting step for NO synthesis, and the sequestration of Arg in intracellular pools that are poorly accessible to eNOS, together with the compartmentalization of eNOS within the caveolae of the plasma membrane, could explain their results [20].

### 2.2. Mechanisms Mediated by Oral Administration of l-Arginine and l-Citrulline on the Vasculature

It is agreed that circulating Arg in the blood, produced by oral Arg or Cit supplementation, may represent a possible therapeutic mechanism to increase the synthesis and bioavailability of NO. The metabolism of orally ingested Arg and Cit differs in some aspects [6]: in contrast to Cit, oral Arg undergoes gastrointestinal and hepatic extraction [21]. Arginases 1 and 2, which are located in the enterocytes of intestines and liver, substantially reduce Arg availability by metabolization to l-ornithine and urea [22]. Nevertheless, Arg supplementation has been reported to improve endothelial dysfunction in humans and animals [23,24]. Cit is not is not acted on by arginases, and skips ‘first-pass’ extraction before it is converted to Arg by argininosuccinate lyase in the kidneys. In addition, the de novo synthesis of Arg from Cit is essential for down-regulating urea formation in the liver to increase nitrogen retention in periods of low protein intake [25]. Thus, it has been shown to be an effective precursor of Arg that serves as a sustained source of Arg in human arteries [22]. Augmenting Arg serves a substrate for the eNOS to produce NO, and thus increase smooth muscle vasodilation. Cit may further directly activate iNOS in skeletal muscle and activated macrophages, as well as indirectly activate nNOS in skeletal muscle, leading to enhanced NO production.

The enzymatic action of eNOS involves a two-step process yielding NO and Cit. It requires Arg as the substrate, as well as the co-substrates O_2_ and reduced nicotinamide adenine dinucleotide phosphate (NADPH), and the cofactors flavin adenine dinucleotide (FAD), flavin mononucleotide (FMN), and tetrahydrobiopterin (BH_4_) [14]. In addition to the presence of the necessary substrates and cofactors, the enzyme must be in its active homodimeric state, which is stabilized by BH_4_ located at the dimer interface [26]. Knowing that Arg serves as the substrate, it is rational to assume that increasing Arg concentrations by pharmacological intervention might be able to improve endothelium-dependent relaxation via increased substrate availability. Indeed, increased concentrations of Arg were reported to improve vascular disease by maintaining NO levels in animal studies [27]. However, since human plasma Arg concentrations rarely reach less than 60 µM even under pathological conditions, and the Michaelis constant, K_m_, for eNOS is merely 3 µM [14], enzyme saturation would be expected, and the administration of excess substrate should not significantly increase NO production. Despite these findings, a linear correlation between plasma Arg concentration and urinary nitrate excretion after the administration of Arg have been demonstrated [28], suggesting that NO production can still be increased by Arg despite a theoretical enzyme saturation, which is a phenomenon referred to as the “l-arginine paradox”.

Several theories trying to explain this phenomenon exist. It has been proposed that the overexpression of endothelial arginases could compete with eNOS for the same substrate [14]. ADMA is the degradation product of methylated proteins and a competitive endogenous inhibitor of eNOS [29]. The enzymatic elimination of ADMA is carried out by dimethylarginine dimethylaminohydrolase, and the activity of this enzyme is inhibited by oxidized Low-density Lipoprotein (LDL) and Tumor Necrosis Factor alpha (TNF-α), as seen in atherosclerosis [30], which would increase ADMA levels and reduce NO production. Besides competitive inhibition, ADMA has also been associated with eNOS uncoupling [31], a state in which the enzyme produces O_2_^–^ instead of NO [14]. Other researchers have proposed that the separation of eNOS and Arg in different intracellular compartments could explain how eNOS is unable to access its substrate despite sufficient total intracellular concentrations. It should be mentioned that other mechanisms of dysfunction unrelated to substrate unavailability exist and include inactivating/activating phosphorylation, the acetylation and glutathionylation of eNOS, protein–protein interactions, and the reactive oxygen species-mediated oxidation of BH_4_ [32].

eNOS activity is regulated by several transcriptional, posttranslational, and physiological factors, affecting NO bioavailability. It can be activated in calcium-dependent or calcium-independent ways [7]. Acetylcholine, bradykinin, and histamine are acting on their specific receptors on the endothelial cell membrane to increase the intracellular concentration of calcium, which binds to calmodulin and induces the activation of the calmodulin-binding domain of eNOS to produce NO [7]. In addition, the phosphorylation of eNOS independently of the calcium concentration is necessary to activate of the enzyme. Thr495 is an inhibitory site, but Ser635 and Ser1179 are activation sites [7]. The responses to hemodynamic shear stress and hormones are mediated mainly through this calcium-independent pathway [7]. The predominant pathway involves the generation of NO from Arg by eNOS and cyclic guanosine monophosphate (cGMP) formation via guanylyl cyclase activation by NO. In vessels, NO is produced mainly from Arg by eNOS, but it can also be released non-enzymatically from S-nitrosothiol or from nitrate/nitrite [7]. The NO production from Arg requires the presence of various cofactors, which are shown in Figure 1. The activity of eNOS and NO production initiated/enhanced by several stimuli and was reviewed in detail by [7]. The eNOS inhibitor ADMA is a known cardiovascular risk factor. Cit supplementation has shown to ameliorate the endothelial function altered by ADMA in porcine coronary arteries [33]. Xuan et al. [33] demonstrated that Cit reversed the down-regulation of the eNOS expression and phosphorylation induced by ADMA. Protein levels of eNOS and p-eNOS-Ser1177 as well as eNOS mRNA level were restored to control values. Ananthakrishnan et al. [34] published in a different study that Cit supplementation ameliorated the development of pulmonary hypertension and increased NO production in piglets exposed to chronic hypoxia. Cit application elevated the NO biosynthesis indirectly by elevating Arg production, resulting in an improved endothelial vasodilator function [6]. Animal investigations support the hypothesis that endothelial function may be ameliorated by the capability of Cit supplementation to increase Arg [19,35].

Taken together, the application of Cit may serve as an attractive non-pharmacological approach for elevating NO bioavailability, which may have the potential to counteract many of the age-related and/or lifestyle-related diseases [36].

## 3. l-Citrulline and l-Arginine as Antihypertensive Compounds

### 3.1. Pharmacokinetics of l-Citrulline and l-Arginine

**Figure 2:**
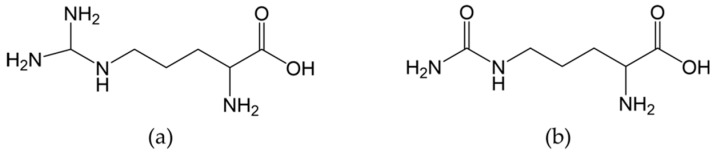
Chemical structure of (**a**) l-arginine and (**b**) l-citrulline.

Arg (Figure 2a) is a smi-essential amino acid found in various foods such as meat and nuts [37]. Based on survey data from 1988–1994, the average American adult intake of Arg was estimated to be 4.40 g/day [38]. Besides dietary intake, de novo synthesis accounts for 5–15% of plasma Arg [39]. Studies investigating the pharmacokinetics of Arg in humans are limited, but data from a 1999 study on 10 healthy volunteers receiving an oral dose of 10 g of Arg showed highly variable bioavailability ranging from 5% to 50% among subjects with an average of 21% [40]. In contrast, a study based on eight healthy males administered an oral dose of 6 g estimated it to 68% [28]. Despite these inconsistent results, the incomplete bioavailability is likely due to considerable first-pass metabolism, as demonstrated in a study using isotopically-labeled tracers of Arg [41]. Clearance under normal circumstances occurs via non-renal mechanisms, with renal clearance taking place when supraphysiological plasma concentrations are achieved, as seen in high-dose intravenous administration, exceeding the kidneys’ reabsorptive capacity [40]. The terminal elimination half-life is 77.5 min for an oral dose of 6 g [28].

Cit (Figure 2b) is a non-essential amino acid that is not commonly found in foods other than watermelon [42]. In contrast to Arg, Cit effectively bypasses the metabolism by the intestine and liver, enabling it to reach the kidneys, where it is converted to Arg [36]. These properties are desirable for increasing systemic Arg concentrations. Schwedhelm et al. investigated how different dosing regiments of oral Arg and Cit affected plasma Arg concentrations (Table 1) and pharmacodynamic parameters such as plasma [Arg]/[ADMA] ratio, urinary nitrate, and cGMP excretion (surrogate marker of NO production) and flow-mediated vasodilation (FMD) after 1 week in 20 healthy volunteers [6]. 

They found that 0.75 g of Cit given twice a day increased the Arg area under the curve (AUC) to the same extent as both 1.6 g of slow release Arg given twice a day, and 1 g of immediate release Arg given three times a day [6], demonstrating the potency of Cit. Only 1.6 g of Arg slow release twice daily and 3 g of Cit twice daily were effective to increase the plasma [Arg]/[ADMA] ratio, and only the latter regiment was successful to elevate the urinary nitrate and cGMP excretion. None of the regiments was able to improve the FMD, and the authors suggest that this finding could be due to the short intervention duration. Interestingly, a correlation between higher FMD changes with an increased [Arg]/[ADMA] ratio was found. The most effective regiment to elevate the [Arg]/[ADMA] ratio was 3 g of Cit twice daily, which was also the only strategy that was able to increase the urinary excretion of nitrate and cGMP. These findings are consistent with this regiment also being the most effective to increase Arg AUC and thus, the total exposure of the endothelium to Arg, suggesting a correlation between plasma [Arg] and these surrogate markers of NO production. This correlation has also been demonstrated earlier by Bode-Böger et al. [28]. The combined use of Arg and Cit has also been proposed as an alternative to the isolated use of either one. Experiments on mice and rabbits receiving oral Cit, Arg, or a combination at a half dosage of each, demonstrate that the combined use was effective at acutely raising plasma Arg levels [24]. A double-blinded, randomized placebo-controlled study on 42 healthy Japanese males randomized to either oral Cit, Arg, a combination at half dosage of each, or placebo had similar results [43]. A later study also found higher mean AUC _0-4 h_ values of plasma Arg for the simultaneous use of Arg and Cit compared to the other dosing regiments, despite not being statistically significant. Interestingly, both papers suggested that the apparent synergistic effect on short-term plasma Arg levels might be due to suppressed arginase activity caused by Cit, as demonstrated by Shearer et al. [44], limiting the first-pass metabolism of Arg. The half-life of Cit has been shown to increase in dose-dependence from 0.65 to 1.14 h for dosages of 2 to 15 g [45].

In summary, the evidence presented above suggests that Cit is more suited for long-term elevations of Arg levels compared to the Arg on a gram-to-gram basis. Furthermore, the simultaneous administration of both compounds might be advantageous in situations where acute increases in plasma Arg are desired.

### 3.2. Results of Clinical Trials

Only very few clinical trials have been conducted to assess the BP-lowering effects of oral Arg administration (Table 2), but a 2011 meta-analysis of randomized, double-blinded, placebo-controlled trials aimed to examine this effect [46]. The analysis included 11 randomized, double-blinded, placebo-controlled trials with a total of 387 participants receiving a median daily dose of 9 g (range: 4 to 24 g/day) for a median of four weeks (range: 2 to 24 weeks). The intervention lowered systolic blood pressure (SBP) by 5.39 mmHg (95% CI: 2.25–8.54, *p* = 0.001) and diastolic blood pressure (DBP) by 2.66 mmHg (95% CI:1.54–3.77, *p* < 0.001) compared to the placebo, and was tainted by little to substantial heterogeneity; *I*^2^ = 73.3% and 34.4% for SBP and DBP, respectively. No statistically significant correlation was found between dose, duration of intervention, or baseline BP on net change in SBP or DBP. Diarrhea was observed in two of the six trials with available data on adverse effects.

It is worth mentioning that in addition to the wide range of intervention durations and dosages, the participants of the individual trials had highly variable baseline BP and health status (healthy, Type 2 diabetic, polycystic ovarian syndrome, hypercholesterolemia, etc.). Interestingly, the majority of participants were normotensive, and the authors speculated that this might have underestimated the effect of the intervention, since the BP of these individuals was already within normal range. As previously mentioned, meta-regression analysis yielded no statistically significant correlation between baseline BP and a reduction in SBP or DBP, but a trend toward greater SBP reduction among participants with higher baseline SBP was observed. The source of heterogeneity was assessed, and seemed to be due to two trials, which after exclusion eliminated the heterogenicity without substantial impact on the final effect. 

In contrast to Arg, no less than three meta-analyses have been published since 2018 on Cit and its effect on BP (Table 3) [47,48,49]. 

Mahboobi et al. [49] pooled data from 15 randomized controlled trials for a total of 424 participants. The trials had to use Cit or Cit-containing food such as watermelon as intervention, and the dosages ranged from 2.7 to 8.4 g/day for 1 to 16 weeks. Fourteen of the trials were placebo-controlled, and nine of the 15 trials were blinded. Reduction in SBP was 7.54 mmHg (95% CI: 5.63–9.44, *p* = 0.0001) and 3.77 mmHg for DBP (95% CI: 1.86–5.67, *p* = 0.0001), with only the latter showing significant heterogeneity (*I*^2^ = 42%). Interestingly, subgroup analyses revealed a larger reduction in both SBP and DBP for study durations ≥6 weeks and baseline BP ≥130/85, suggesting that Cit more effectively reduces BP with long-term administration and when given to pre-hypertensive and hypertensive individuals. Counterintuitively, the subgroup analysis also revealed that doses ≤4 g/day was more effective at reducing both SBP and DBP. None of the 15 trials reported any adverse side effects.

As in the case of the meta-analysis by Dong et al. on Arg [46], the health status, baseline BP, dosages, and intervention durations varied considerably between trials. Furthermore, the authors did not elaborate on five of the 15 trials using watermelon extract as intervention, which contains both Cit and Arg in the ratio of 2:1. As previously mentioned, the current evidence suggests that Arg exerts a BP-lowering effect, which means that the intervention effect found by Mahboobi et al. [49] may not solely be attributed to Cit, as Arg may very likely have confounded the results.

A similar meta-analysis as the above mentioned was conducted by Barkhidarian et al. [48]. They pooled data from randomized controlled trials examining the effect of Cit on BP, but in contrast to Mahboobi et al. [49], they excluded trials using Cit mixed with other substances. Thus, all trials using watermelon as the source of Cit were excluded. Their data consisted of 190 subjects from eight trials, all of which were also used in the analysis done by Mahboobi et al. [49]. Cit supplementation reduces both SBP and DBP, with only SBP being significant; 4.10 mmHg (95% CI: 0.26–7.94, *p* = 0.037) and 2.08 mmHg (95% CI: −0.16–4.32, *p* = 0.069). A subgroup analysis on dosages ≥6 g/day later revealed a significant reduction in DBP: 2.75 mmHg (95% CI: 0.12–5.37, *p* = 0.04).

The effect of Cit in this analysis is not influenced by confounding from Arg, but is otherwise subject to the same issues regarding the characteristics of the participants. The overall results suggest that Cit can significantly reduce BP, with a reduction in DBP only being significant with higher dosages. The inability to demonstrate significant DBP reductions without subgroup analysis might be due to the small sample size.

A third meta-analysis by Mirenayat et al. [47] suggests that Cit does not have beneficial effects on blood pressure. The inclusion and exclusion criteria were similar to those of Barkhidarian et al. [48], and therefore excluded any trials using Cit from watermelon extracts. Six studies were initially identified, with one of them later being excluded from the analysis because it reported pulmonary BP. Thus, their data included 114 subjects from five trials, all of which were also included in the analysis by Barkhidarian et al. [48]. A further comparison of the trials revealed that three trials [51,52,53] included by Barkhidarian et al. [48] were not included in the analysis by Mirenayat et al. [47]. A comparison of the search strategy and study selection did not explain the apparent discrepancy. All three trials [51,52,53] demonstrated a lowering effect on BP, and the failure to include these trials might explain the findings of Mirenayat et al. [47]. A methodical difference worth mentioning is that Mirenayat et al. [47] differentiated between changes in aortic and brachial BP, and found no significant change for either one.

## 4. Discussion

Hypertension is a well-known risk factor for cardiovascular disease, and is attributable to almost 10 million deaths yearly worldwide [4]. Much research has been conducted to understand the underlying pathophysiology of essential hypertension, which accounts for 95% of cases [2]. Both human and animal studies have suggested that dysfunctions in the NO pathway may play a central role in the pathogenesis of hypertension. The regulation of the pathway is complex, and substrate inaccessibility has been investigated as a potential target for pharmacologic intervention, but no definitive theory exists for the apparent “l-arginine paradox”. Basic science on the pharmacokinetic properties of Arg and Cit show that Cit is more effective at increasing Arg levels, which is the main proposed mechanism by which these compounds exerts their BP-lowering effects. Several small clinical trials have been conducted for both compounds. Although current evidence is sparse, it suggests a significant BP-lowering effect of Arg: 5.39/2.66 mmHg. To put these numbers into perspective, the clinical significance of these results should be compared to those obtainable with lifestyle modifications such as healthy diet and aerobic exercise. Implementation of the Dietary Approaches to Stop Hypertension (DASH) diet has been shown to exert similar BP-lowering effects (6.74/3.54 mmHg [54]), and less pronounced effects for aerobic exercise (3.84/2.58 mmHg) [55]. Furthermore, most clinicians would agree that prescribing oral supplements is adventitious in terms of patient compliance compared with rather comprehensive lifestyle modifications such as diet restriction and exercise. Since Arg is a naturally occurring amino acid with minor reported side effects such as diarrhea, one might also expect certain patients to favor it over more traditional and “unnatural” antihypertensive drugs such as beta-blockers and Angiotensin Converting Enzyme (ACE) inhibitors, and thus limit unnecessary pathologization.

There is currently no consensus regarding the efficacy of Cit in lowering BP, as evident from three recently published meta-analyses [47,48,49]. The inability to reach an accordant conclusion seems to be due to methodological differences in inclusion and exclusion criteria and the identification of suitable trials. Still, two [48,49] of the three papers suggest a BP-lowering effect that is clinically comparable with Arg. It is worth mentioning that the validity of the analysis by Mirenayat et al. [47], who did not find an effect, should be questioned. This review identified three trials that were missing in the analysis by Mirenayat et al. [47] based on the presented study criteria and search strategy, which could very likely have changed the outcome of the analysis. Taking this into consideration, a BP-lowering effect of Cit is very likely, and is coherent with its established pharmacokinetic properties and proposed mechanism of action. The same considerations regarding patient compliance and pathologization would hold true for Cit as for Arg, with Cit having an even greater safety profile, as none of the trials reported any adverse effects.

## 5. Conclusions and Outlook

Endothelium-dependent vasodilation via NO is essential for cardiovascular regulation in normal human physiology. Dysfunction of the NO pathway occurs via several mechanisms, including substrate unavailability, and has been investigated as a potential pharmacological target. The administration of NO precursors in the form of Arg have shown promising results with less convincing results for Cit, despite its theoretically advantageous pharmacokinetic properties. Their ease of administration and sparse adverse effects makes them great candidates in the first-line treatment of borderline or mild hypertension in addition to lifestyle changes, but more research is needed to determine their efficacy and safety as antihypertensive compounds.

Current evidence suggests a BP-lowering effect of Cit and Arg, but more research is needed to solve the “l-arginine paradox” and understand the exact mechanism by which this occurs. The BP-lowering effect found in the above-presented meta-analyses for both Arg and Cit is based on several small trials with heterogenic study populations regarding baseline BP and comorbidity, in which highly variable intervention durations and dosages were used. More research with larger study populations is needed to confirm these observed effects, establish optimal dosing regiments, and identify possible adverse effects. Furthermore, it would be interesting to identity possible effect modifiers, such as age, gender, comorbidity, and baseline BP, with the latter already demonstrated [49]. This knowledge would be a valuable tool for both the clinician and patient when determining whether one is likely to benefit from these compounds.

## Figures and Tables

**Table 1:** Plasma l-arginine area under the curve (AUC) after 1 week of different l-citrulline and l-arginine dosing regiments [6].

Compound	Dose (mg) × Number of Daily Administrations	Plasma [arginine] AUC (μmol·h·L^−1^) ± SEM
l-citrulline	750 × 2	271 ± 38
lL-citrulline	1500 × 2	421 ± 65
lL-citrulline	3000 × 2	898 ± 67
lL-arginine slow release	1600 × 2	289 ± 50
lL-arginine immediate release	1000 × 3	283 ± 51

**Table 2:** A list of clinical trials investigating the efficacy of l-arginine on hypertension (https://clinicaltrials.gov), last accessed on 12 July 2019.

Title and trial identifier	Objective	Design	Status and results
l-arginine treatment in mild hypertension (LAHN) NCT02894723	To evaluate the efficacy of Arg treatment on blood pressure control patients with stage 1 hypertension	Dietary Supplement: Arg Dietary Supplement: syrup Patients will receive Arg 30 mL twice a day for eight weeks. Phase 4	Not yet recruiting
l-arginine effects on chronic hypertension in pregnancy NCT00974714	To evaluate the effects of oral Arg administration on pregnant women at second trimester of gestation with chronic hypertension, with respect with placebo.	Oral Arg 2 g twice a day, for 14 weeks Placebo-controlled Phase 3	Completed (2010)
l-Arginine metabolism in essential hypertension NCT00137124	This study determines whether metabolism and transport of Arg are altered in patients with essential hyper-tension and whether these potential alterations can be targeted therapeutically.	Interventional, randomized trial with 120 participants, oral administration of Arg for 4 weeks Phase 2	Completed (2009)
Effects of oral l-arginine on chronic hypertension in pregnancy NCT00571766	To evaluate the effects of oral Arg in pregnant women with chronic hypertension.	Interventional (Clinical Trial), randomized with 80 participants, oral Arg 2 g twice a day for 14 weeks Phase 3	Completed (2008)
Effect of l-arginine and pycnogenol on light to moderate hypertension and endothelial function NCT02392767	To evaluate the effect of a combination product (Verum) with Arg, Pycnogenol, vitamin K2, R-(+)-alpha-lipoic acid, and vitamins B6, B12, and folic acid	A randomized, double-blind, placebo-controlled cross-over study Two tablets twice a day for four weeks. Verum/Placebo	Completed (2015) Systolic blood pressure decreased significantly under the Arg-based multi-ingredient product (AbMIP) [50]
Impact of citrulline and arginine supplementation on the post-exercise hypotension (PEH) NCT03378596	To increase the knowledge regarding non-pharmacological models aimed at the prevention and treatment of hypertension in normotensive and hypertensive patients. Cit (6 g) Arg (8 g)	Interventional (clinical trial), randomized, 20 participants, ambulatorial blood pressure monitoring	Recruiting
Effects of inhibition of NO synthesis on renal hemodynamics and sodium excretion in patients with essential hypertension and healthy controls NCT00345150	To test the hypothesis that systemic and renal nitric oxide synthesis is changed in essential hypertension by investigating the effects of a non-selective nitric oxide inhibitor on renal hemodynamics and sodium excretion in patients with essential hypertension. N^G^-monomethyl- l-arginine	Interventional (clinical trial), randomized, 30 participants Phase 1	Completed (2006)
l-arginine, vascular response and mechanisms NCT01482247	To employ the supplement Arg to test the hypothesis that the activation of blood flow to the brain during cognitive tasks is regulated by nitric oxide in older subjects with diabetes mellitus and/or hypertension. Arg as supplement	Interventional (Clinical Trial), randomized, 25 participants. Phase 2	Completed (2014)

**Table 3:** Summary of the results of meta-analysis investigating the effects of oral l-citrulline and l-arginine supplementation on blood pressure.

Author and Publication Year	Supplement	Total Number of Trials and Participants	Reduction in SBP	Reduction in DBP	Dose and Duration
Dong et al. 2011 [46]	l-arginine	11 trials 387 participants	5.39 mmHg (95% CI: 2.25–8.54, *p* = 0.001)	2.66 mmHg (95% CI:1.54–3.77, *p* < 0.001)	2–24 weeks 4–24 g/day
Mahboobi et al. 2019 [49]	l-citrulline or watermelon extract	15 trials 424 participants	7.54 mmHg (95% CI: 5.63-9.44, *p* = 0.0001)	3.77 mmHg for DBP (95% CI: 1.86–5.67, *p* = 0.0001)	1–16 weeks 2.7–8.4 g/day
Barkhidarian et al. 2019 [48]	l-citrulline	8 trials 190 participants	4.10 mmHg (95% CI: 0.26–7.94. *p* = 0.037)	2.08 mmHg (95% CI: −0.16–4.32. *p* = 0.069)	1–17 weeks 3–9 g/day
Mirenayat et al. 2018 [47]	l-citrulline	5 trials 114 participants	Brachial: 0.28 mmHg (95% CI: −2.87 to 2.31) Aortic: 0.22 mmHg (95% CI: −4.81 to 4.38)	Brachial: −1.56 mmHg (95% CI: −4.30 to 1.20) Aortic: 0.26 mmHg (95% CI: −2.27 to 2.80)	1–8 weeks 3–6 g/day
